# Clinical Holistic Medicine: Developing from Asthma, Allergy, and Eczema

**DOI:** 10.1100/tsw.2004.191

**Published:** 2004-10-28

**Authors:** Søren Ventegodt, Mohammed Morad, Joav Merrick

**Affiliations:** ^1^ The Quality of Life Research Center, Teglgårdstræde 4-8, DK-1452 Copenhagen K, Denmark; ^2^ Clalit Health Services and Division of Community Health, Ben Gurion University, Beer-Sheva, Israel; ^3^ National Institute of Child Health and Human Development, Office of the Medical Director, Division for Mental Retardation, Ministry of Social Affairs, Jerusalem and Zusman Child Development Center, Division of Pediatrics and Community Health, Ben Gurion University, Beer-Sheva, Israel

**Keywords:** quality of life, QOL, philosophy, human development, holistic medicine, public health, holistic health, holistic process theory, life mission theory, asthma, allergy, eczema, psychosomatics, complementary and alternative medicine, Denmark, Israel

## Abstract

This paper shows how consciousness-based holistic medicine can be used in the case of asthma, allergy, and eczema. We have many fine drugs to relieve patients from the worst of these symptoms, where many children and adults suffer health problems related to hyper-reactivity of the immune system. Many symptoms remain throughout life because the drugs do not cure the allergy and allergy today is the sixth leading cause of chronic illness. The etiology of the immune disturbances is mostly unknown from a biomedical perspective. Consciousness-based holistic medicine could therefore be used to treat these diseases if the patient is willing to confront hidden existential pain, is motivated to work hard, and is dedicated to improve quality of life, quality of working life, and personal relationships. Improving quality of life is not always an easy job for the patient, but it can be done with coaching from the physician. An increased physical health is often observed after only a few sessions with a physician skilled in using holistic medical tools and able to coach the patient successfully through a few weeks of dedicated homework. Children with allergy and asthma can also be helped if their parents are able to do work on personal development, to improve the general quality of life in the family and their relationship with the child.

## INTRODUCTION

Asthma, allergy, and eczema are believed to have a psychosomatic dimension[1,2,3], which can be understood due to the fact that many children and adolescents who have asthma, allergy, or eczema grow out of it. This is very fortunate because many modern-day children suffer from allergies[4]. Each year, more than 50 million Americans suffer from allergic diseases and allergies are the sixth leading cause of chronic disease in the U.S., costing the healthcare system $18 billion annually[4]. Estimates of allergy prevalence in the U.S. were 9–16%, with the prevalence of allergic rhinitis increased substantially over the past 15 years[4]. Approximately 16.7 million office visits to healthcare providers each year are attributed to allergic rhinitis[4]. Since many children, adolescents, and adults are afflicted with allergies, it is worth looking at "what growing out of it" means and how this process of spontaneous healing can be supported.

Asthma, allergy, and eczema are diseases that are associated with some minor disturbances in the immune system. In allergy, the body attacks foreign antigens (substances that are alien to the body) even if they are not in any way harmful to the body. So when the immune system defends itself against pollen that penetrates down into the lungs, this may result in allergic asthma; in the nose, it can cause hay fever (allergic rhinitis) and on the skin, allergic eczema. Very often, there is nothing clearly allergic in eczema, even though we know that it is generally associated with allergy. When the skin is weak, this is not due to chance, from a holistic point of view, but to the work of the cells in the skin that usually make the skin thick, strong, and healthy, disturbed due to blockages contained in it.

When we grow out of our disease, it is because during development we are confronted with many of the things that disturb our inner balance and in the process the blockages are lifted. When we sometimes do not grow out of diseases, it is because we have managed to avoid confronting our earlier emotional difficulties. The holistic physician will support the patient in confronting the problems beneath the surface that are the cause of the disease from a holistic perspective.

## CLINICAL HOLISTIC MEDICINE

The life mission theory[5,6,7,8,9,10] states that everybody has a purpose of life or a huge talent. Happiness comes from living this purpose and succeeding in expressing the core talent in your life. To do this, it is important to develop as a person into what is known as the natural condition, a condition where the person will get to know himself and use all his efforts to achieve what is most important for him. The holistic process theory of healing[11,12,13,14] and the related quality of life theories[15,16,17] state that the return to the natural state of being is possible whenever the person gets the resources needed for the existential healing. The resources needed are "holding" in the dimensions awareness, respect, care, acknowledgment, and acceptance with support and processing in the dimensions feeling, understanding, and letting go of negative attitudes and beliefs. The precondition for holistic healing to take place is trust and the intention for the healing to take place. Existential healing is not a local healing of any tissue, but a healing of the wholeness of the person, making him much more resourceful, loving, and knowledgeable of himself, his own needs and wishes. In letting go of negative attitudes and beliefs, the person returns to a more responsible existential position and an improved quality of life. The philosophical change of the person healing is often a change towards preferring difficult problems and challenges, instead of avoiding difficulties in life[18,19,20,21,22,23,24,25]. The person who becomes happier and more resourceful often also becomes more healthy, more talented, and more able to function[26,27,28].

## CASE REPORTS

The following case stories are all about adults, but we believe that children can be helped in a similar way[29]. Allergy has been on the increase and it afflicts millions of people in the Western world[4], but we have seen that it can be treated with consciousness-based medicine if the patient is willing to look at her/himself and life honestly. It is quite remarkable and enlightening to experience how a physical ailment can be slowly transformed into the mental and emotional ailments underlying it. The patient in the case below did really well and was rewarded almost immediately by ridding herself of the allergy.

> **Female, aged 29 years, with allergy:** First visit: Auscultation of the lungs: Among other things, highly stressed. She has been coughing for eight days, severe headaches a few days ago, her eyes are very sore with allergy, also has problems with her former partner, who does not see their joint child regularly. We talk a little about the patient's apparent terror of illness and doctors. She can come for conversation on allergy etc., if she wishes to do so.
>
> Second visit: Conversation: The patient talks about the imbalances in her life that she would like to correct: "I never feel that I am" – the being/doing imbalance; "more brain than heart" feeling/thinking imbalance and give/take imbalance: "I always have to give, so my new boyfriend will have to give to me" – she is critical, demanding, controlling instead of loving and accepting. She has family and work, but no leisure time. EXERCISE: She will look at that for next time.
>
> Third visit: The allergy is getting better. She comes with four written pages, which she reads out, but does not want to hand over, as she feels vulnerable. That is fine, she has done her homework. We talk about her vulnerability. She feels abandoned, alone, demeaned, naked and small in it. She therefore sets aside her emotions; she associates those with weakness and vulnerability, which she does not like. We talk about her superficiality, and as an EXERCISE for next time she is to draw up a list of her qualities as a beautiful, good and true individual and a list of the losses in her life, where she gave up being that kind of an individual The project is rehabilitation of her emotions. She is pleased with the way things have gone.
>
> Fourth visit: Conversation. We talk about the patient having gone to extremes in being active (high tempo, action), controlling, "masculine", remote and superficial. She describes her relations with men as "non-serious" and her relationships as "empty" - but life itself has lots of content. We talk about the possibility of the activity being a defence against a deeper pain, which she is running away from. EXERCISE: Sit and be, feel your emotions, let go of body and thoughts. 5 – 10 – 15 – 20 min every day, preferably increasing slowly, for example weekly.

The personal problems will take her years to solve and she will probably not avoid having a prolonged course of gestalt therapy, or something similar, if she wants to entirely rid herself of the feeling of being "abandoned, alone, demeaned, naked and small". We again see that as soon as the patient starts on her exercises, works purposefully, and under precise guidance on herself at home and in her everyday life, things move quickly and effectively. Note also the severe pain suffered by the patient in the process. These pains come about when the patients look at themselves and acknowledge how things really are. Many patients are not at all willing to feel small, alone, humiliated, abandoned, the feeling of being naked, because being looked through one's suffering is quite simply terrible — embarrassing and shameful — for many people. There is therefore good reason to carry on living with the allergy you received.

> **Female, aged 44 years, with burning sensation in joints:** Physiotherapy helps. The therapist writes that development of the patient's overview and understanding of the situation is going well. There is "burning" in the body and joints. On examination:

> Tender and tense muscles in legs, thighs and back. We talk about the sadness of life. Ought to be moving on, but where to? EXERCISE 1: By next time, the patient must learn to accept her emotions of sadness, she should ideally sit on a bench for five to ten minutes and be as sad, as she can when the sadness comes over her. Divorced ten years ago with small children and that was difficult. The eldest two have left home, only a boy of ten is left. EXERCISE 2: Make a good plan for your new life. What do you wish for yourself? How can you obtain it? Prescribe continued physiotherapy.

In this case, we also looked for a form of allergy: spontaneous "burning" or rather a feeling of inflammation in the body, which is due to the tissues that hold on to the repressed emotions being able to react with inflammation. Inflammation is a way of the cells to shout "help, there is something wrong here!" The signal is often mediated by histamine, which several types of cell can secrete (e.g., mast cells). An antihistaminergic drug (e.g., Zyrtec [cetirizine]) can therefore subdue the symptom. We naturally prefer a causal cure.

> **Female, aged 38 years, Will she do something herself?:** 1. Complain of urticaria. No visible patches. 2. Low-back pain. On examination: muscle tension between the shoulder blades, tender trigger points corresponding to the low back (sacro-iliac joints), tender over left knee, thinks herself that she has food allergies and arthritis. There is nothing to suggest this. I tell the patient this. I am in doubt as to whether the patient wishes to do something herself for her own health. This patient tells me that she has arthritis and food allergy. Bent Weeke, specialist in allergy at Copenhagen University Hospital, carried out a study, where he gave around 150 patients what they could not tolerate as "astronaut food", that is to say in a tube[29]. They did not know what was in it. Without being aware that they were actually receiving the very substance they could not tolerate, they all, apart from two, were able to tolerate the food to which they were "allergic". This study showed that food allergy in 98.5% of the cases is not allergy or hypersensitivity in the traditional biological sense, but something else, which relates to the mind. When a patient tells me that she has a food allergy, we therefore know immediately that there is a less than 2% probability that this is correct. She did not have arthritis or urticaria (nettle rash) either.

So there is a clear somatization of a psychological or existential problem here. Her problem can be solved, but she first has to acknowledge her "real disease", that is to say the problems, which she somatisizes. She was not motivated for that. So what can be done? We cannot medicate her for diseases she does not have and we cannot just ignore her when she comes looking for help. In reality, she is stunningly indifferent towards our professional advice, she already knows what is wrong with her, so just treat that, if you please! Incredible! Even the greatest capabilities are insufficient to deal with this. We have to ask her to please find another physician with greater expertise.

> **Female, aged 58 years, with obsessive-compulsive neurosis, allergy unlikely:** Quality-of-life conversation: The patient has a history of autoimmune myxoedema treated with Eltroxin [levothyroxin]. She is well controlled according to test results reported by her own physician. Had a heavy fall nine years ago while skiing with subsequent back problems. A few months afterwards she fell backwards off a step-ladder, eight years ago myelography, subsequent complications in the form of persistent headache, for months afterwards hearing problems and impaired vision, which the patient still has, in addition the patient had allergy with stinging pain in the digestive system, "food allergy", established by specialist in allergy, apparently does not tolerate chemical things, moulds and sprays, curry, chilli, oregano, pills in general. In recent years constant pain in joints and muscles, spontaneous fracture in the knee last year – one month in cast. The patient has always been hypermobile. Has previously gone from doctor to doctor without

> success, has also tried alternative medicine, like attending a nutrition clinic for three to four years and has been given a dietary supplement without any beneficial effect. The patient is at present claiming compensation from an insurance company. The assessment is a patient in a poor condition physically, mentally and also in need of extensive holistic rehabilitation. The patient insists that a number of perspectives and matters are of importance and totally true. It seems that the problem is a move of her life energy out of her body and into her head. /suspected obsessive-compulsive neurosis/. PLAN: She should have gestalt therapy, where a treatment plan is to be drawn up. EXERCISE 1: Fill about four pages of A4 paper with afflictions by next time – list all the problems in life: social, physical, sexual, financial, spiritual etc. EXERCISE 2: Also draw up a detailed list of your wishes.

We feel that this patient experienced discomfort with all the things mentioned and we did not believe that the correct diagnosis is “allergy”. We believe that she has a serious mental disorder and conclude with a tentative diagnosis of “obsessive-compulsive neurosis”. Genuine food allergy, as mentioned above, is extremely rare.

## DISCUSSION

To explain the etiology of the disturbances in the regulations of the immune system, we need to understand the connection between consciousness and the cellular order of the body. As consciousness is almost a complete mystery for biochemical science[30,31], we have to wait a while to get the full explanation, which obviously must be presented, if we are to integrate biomedicine and consciousness-based medicine. But having two or even three different paradigms — the manual, the biomedical, and the holistic — with a similar number of toolboxes and approaches, works really well for us. The depth of existence and nature is really overwhelming, but this should not prevent us from doing well as medical doctors. Sometimes we prescribe a drug to a businessman not motivated for spending many hours contemplating the meaning of his life; sometimes we give an exercise like “write your biography” to patients in a period without a job, allowing them to use the precious time of their life on themselves, improving not only their health, but also their general quality of life, ability to function, and expression of their talents, thus preparing them for a fine comeback to professional life. To be dynamic, flexible, and receptive as physicians seems to be of utmost value to our patients.

Asthma, allergy, and eczema can often be cured with consciousness-based holistic medicine. A high percentage of our children and almost as many adults suffer from such health problems related to the immune system[4]. We have drugs that can relieve the patient from the worst of these symptoms, but the problems often remain throughout life, as a chronic disease. The etiology of the immunological disturbances, i.e., the hyper-reactivity-related diseases, which have been the focus of this paper, are mostly unknown from a biomedical perspective. Turning to consciousness-based holistic medicine seems quite simple to cure these diseases if the patient is willing to confront hidden existential pain, is motivated to work hard, and is dedicated to improve the quality of life, quality of working life, and personal relationships. This is not a small task, but can be done over time and the improvement of the symptoms are often noticed after only a few sessions with a physician skilled in using holistic medical tools and able to coach the patient successfully through a few weeks of dedicated homework. Children can also be helped[32] if their parents are willing to do work on personal development, to improve the general quality of life in the family and their relationship with the child. We often see that the child’s quality of life and health status from the perspective of holistic medicine often is a thermometer for the thriving of the whole family.

## ACKNOWLEDGMENTS

This study was supported by grants from IMK Almene Fond. The quality of life research was approved by the Copenhagen Scientific Ethical Committee under number (KF)V.100.2123/91.

## BIOSKETCHES

**Søren Ventegodt, MD,** is the Director of the Quality of Life Research Center in Copenhagen, Denmark. He is also responsible for a Research Clinic for Holistic Medicine in Copenhagen and is a popular speaker throughout Scandinavia. He has published numerous scientific or popular articles and a number of books on holistic medicine, quality of life, and quality of working life. His most important scientific contributions are the comprehensive SEQOL questionnaire, the very short QoL5 questionnaire, the integrated QOL theory, the holistic process theory, the life mission theory, and the Danish Quality of Life Research Survey, 1991–94 in cooperation with the University Hospital of Copenhagen and the late pediatric professor Bengt Zachau-Christiansen. E-mail: ventegodt@livskvalitet.org. Website: http://www.livskvalitet.org

**Mohammed Morad, MD**, is Specialist in Family Medicine, Lecturer in Family Medicine at the National Institute of Child Health and Human Development, Division of Community Health, Ben Gurion University of the Negev and the Medical Director of a large area clinic in the city of Beer-Sheva. He has publications on Bedouin health, health aspects, spiritual health, and aging in persons with intellectual disability, and is a presenter on topics such as health policy and services for the disadvantaged at national and international conferences. E-mail: morad62@barak-online.net

**Joav Merrick, MD, DMSc,** is Professor of Child Health and Human Development affiliated with the Zusman Child Development Center, Division of Pediatrics and Community Health at the Ben Gurion University, Beer-Sheva, Israel; the Medical Director of the Division for Mental Retardation, Ministry of Social Affairs, Jerusalem; and the Founder and Director of the National Institute of Child Health and Human Development. He has numerous publications in the field of child and human development, rehabilitation, intellectual disability, disability, health, welfare, abuse, advocacy, quality of life, and prevention. Dr. Merrick received the Peter Sabroe Child Award for outstanding work on behalf of Danish Children in 1985 and the International LEGO-Prize ("The Children's Nobel Prize") for an extraordinary contribution towards improvement in child welfare and well being in 1987. E-mail: jmerrick@internet-zahav.net. Website: www.nichd-israel.com
